# A mixed-methods systematic review of suicide prevention interventions involving multisectoral collaborations

**DOI:** 10.1186/s12961-022-00835-0

**Published:** 2022-04-14

**Authors:** Tania Pearce, Myfanwy Maple, Sarah Wayland, Kathy McKay, Alan Woodward, Anna Brooks, Anthony Shakeshaft

**Affiliations:** 1 School of Health, University of New England, Armidale, NSW 2351 Australia; 2 Public Health, Policy and Systems, Institute of Population Health, University of Liverpool, Liverpool, United Kingdom; 3 Tavistock and Portman NHS Foundation Trust, London, United Kingdom; 4 Centre for Mental Health, School of Population and Global Health, University of Melbourne, Melbourne, VIC 3010 Australia; 5 Lifeline Research Foundation, Canberra, ACT 2601 Australia; 6 National Drug and Alcohol Research Centre, University of New South Wales, Randwick Campus, 22-32 King Street, Randwick, NSW 2031 Australia

**Keywords:** Suicide prevention, Multisectoral collaborations, Stakeholder, Consumers, Co-creation, Co-ideation, Co-design, Co-implementation, Co-evaluation

## Abstract

**Background:**

Governments and third-sector organizations (TSOs) require support to reduce suicide mortality through funding of suicide prevention services and innovative research. One way is for researchers to engage individuals and services in multisectoral collaborations, to collaboratively design, develop and test suicide prevention services and programmes. However, despite widespread support, to date, it remains unclear as to the extent to which stakeholders are being included in the research process, or if they are, how these partnerships occur in practice. To address this gap, the authors conducted a systematic review with the aim of identifying evidence of multisectoral collaborations within the field of suicide prevention, the types of stakeholders involved and their level of involvement.

**Methods:**

The authors conducted a strategic PRISMA-compliant search of five electronic databases to retrieve literature published between January 2008 and July 2021. Hand-searching of reference lists of key systematic reviews was also completed. Of the 7937 papers retrieved, 16 papers finally met the inclusion criteria. Because of data heterogeneity, no meta-analysis was performed; however, the methodological quality of the included studies was assessed.

**Results:**

Only one paper included engagement of stakeholders across the research cycle (co-ideation, co-design, co-implementation and co-evaluation). Most stakeholders were represented by citizens or communities, with only a small number of TSOs involved in multisectoral collaborations. Stakeholder level of involvement focused on the co-design or co-evaluation stage.

**Conclusion:**

This review revealed a lack of evidence of multisectoral collaborations being established between researchers and stakeholders in the field of suicide prevention research, even while such practice is being espoused in government policies and funding guidelines. Of the evidence that is available, there is a lack of quality studies documenting the collaborative research process. Also, results showed that the inclusion of co-researchers from communities or organizations is defined as co-creation, but further analysis revealed that collaboration was not consistent across the duration of projects. Researchers and practitioners should consider issues of power and equity in multisectoral collaborations and encourage increased engagement with TSOs, to rigorously research and evaluate suicide prevention services.

## Background

With suicide remaining the leading cause of death worldwide [1], there is growing policy interest by both governments and health agencies in multisectoral collaborations as an innovative approach to suicide prevention [2, 3]. Multisectoral collaborations are characterized as a “bottom-up participatory process” where partnerships are formed between governments, third-sector organizations (TSOs), community members, citizens and researchers to address social issues [2]. While the concept of multisectoral collaborations is not new [2], they are increasingly seen as integral to addressing complex problems such as mental health and suicide prevention [2]. For instance, suicide prevention policies currently promote the establishment of multisectoral collaborations between service providers, consumers with lived experience and researchers, who are then charged with the design, delivery and evaluation of suicide prevention activities [4]. Multisectoral collaborations have the potential to improve the quality of service delivery and the quality of research being produced [5]. These types of improvements can be accomplished by ensuring that the services themselves, and the application of high-quality research methods, are highly tailored to local circumstances [6–8], along with advances in the understanding of co-creation of new knowledge [9]. Evidence for the policy and practice of multisectoral collaborations in suicide prevention originates overwhelmingly from high-income countries (HICs) such as Australia, the United States and Canada, with less evidence coming from low- and middle-income countries (LMICs) where suicide rates remain high, such as Bangladesh [10]. Although LMICs do engage in multisectoral collaborations, issues of underreporting and ineffective data collection systems influenced by sociocultural beliefs about suicide have hindered the development of suicide prevention programmes, thus limiting the creation of multisectoral collaborations [11].

Across both HICs and LMICs, approaches used by researchers working in multisectoral collaborations include the use of participatory research along with knowledge translation models such as integrated knowledge translation (IKT) [12] and frameworks including co-creation of new knowledge [9]. These research models and frameworks are geared towards the formation of multisectoral collaborations between researchers and end-users where the end-user is central to the design and creation of new health programmes or products [13]. When engaging with researchers it is expected that end-users, along with other stakeholders, will be equitably involved across the research continuum from problem definition through to evaluation and dissemination [14, 15]. It is thought that equal levels of engagement by stakeholders including end-users may lead to more effective interventions and improved service and health outcomes [16]. However, achieving equality in multisectoral collaborations is dependent on the type of approach used [17] and the amount of power citizens and community have over the research process [16]. Participants’ level of involvement as informants or consultants means they have little impact on service, intermediate or health outcomes [18], while increased levels of engagement lead to greater empowerment and have a greater impact on outcomes [19]. Divisions in knowledge and experience between stakeholders can also lead to a perceived imbalance of power, reducing levels of stakeholder participation, impeding the production of innovative ideas [20] and eroding trust among participants [21]. According to Dillon [22], power in participatory research approaches manifests as “both the external power relations that influence researchers and stakeholders and in the collaborative actions of the project”. Ignoring the power dynamics that inevitably exist in multisectoral collaborations can be detrimental to the research cycle on a whole, particularly with regard to the methodological quality of studies [16]. Generally, the quality of methodological evidence of effective suicide preventative interventions in conventional positivist research remains weak [23]. Strong collaboration between researchers and stakeholders offers the opportunity for democratic discussions about research design, recruitment of participants, data collection methods and discussion of empirical findings, thus increasing the likelihood of quality evidence [16]. While this approach demonstrates promise in improving suicide prevention outcomes, our understanding of the scale of multisectoral collaborations and the range and extent of engagement and participation in these collaborations remains unclear.

It is important for the field of suicide prevention to consider the potential contribution multisectoral collaborations may have on advancing the quality of suicide prevention research by synthesizing and critically reviewing existing studies. Further to this, evaluating the strength and methodological quality of multisectoral collaborations will provide awareness on the efficacy of multisectoral collaborations in improving the evidence base. To the best of the authors’ knowledge, no previous systematic reviews have comprehensively examined the extent and nature of multisectoral collaborations in the field of suicide prevention. Systematic reviews published in the last 5 years have investigated multisectoral collaborations between researchers and stakeholders in the broader context of mental health, but nothing specific to suicide prevention. A review of 16 studies on consumer involvement in the co-design of mental health interventions, for instance, found little evidence of equitable collaboration between stakeholders. In these cases, stakeholder involvement of young people as end-users was limited to the role of advisor or consultant in the co-design of effective technology-based mental health interventions including mobile phone apps and text messaging services [24]. Similarly, a more recent systematic review of 20 papers on the involvement of mental health service users and their caregivers in practice and policy in LMICs found service users assigned to the role of study participants and less likely to be involved in the direct planning and development of programmes and services [25]. In this study, evidence of collaborations between service providers, mental health professionals, and caregivers and/or service users was associated with a poor evidence base. To address gaps in our knowledge, we conducted a systematic review aiming to examine the current state of evidence on multisectoral collaborations in suicide prevention research implemented globally. Based on the current body of evidence, we sought to (1) identify and describe multisectoral collaborations focused on suicide prevention interventions and/or their development, (2) explore the level of involvement and how these impact on power relationships between researchers and stakeholders, including issues of inequality, (3) explore the outcomes of multisectoral collaborations across the research cycle and (4) evaluate the methodological quality of preventative interventions.

The aims and objectives of the systematic review were formulated in conjunction with the research team, most of whom possess diverse experiences with providers and consumers of mental health services, with lived experience of suicide, and as researchers.

## Operational definitions

Operationalized concepts and definitions of key terms used throughout this paper including multisectoral collaborations, co-creation, suicide prevention research, stakeholder, TSOs, level of involvement and outcomes are listed in Table 1.

**Table 1:** Operational definitions

Term	Definition
Collaboration	An interactive process that enables people with diverse expertise to generate creative solutions to mutually defined problems [75]. In this paper, partnership is used interchangeably with collaboration
Co-creation	The generation of new knowledge that is derived from the application of rigorous research methods that are embedded in the delivery of a programme or policy (by researchers and a range of actors including service providers, service users, community organizations and policy-makers) through four collaborative processes: (i) generating an idea (co-ideation), (ii) designing the programme or policy and the research methods (co-design), (iii) implementing the programme or policy according to the agreed research methods (co-implementation), and (iv) the collection, analysis and interpretation of data (co-evaluation) [9]
Lived experience	“Lived experience” refers to the direct experience a person has of states of distress commonly labelled as “mental illness”, and it also refers to experiences with using mental health services, or not being able to access them [76]
Multisectoral collaboration	Multiple sectors and stakeholders intentionally coming together and collaborating in a managed process to achieve shared outcomes [77]
Suicide prevention research	Activities which collect new data or carry out some novel analysis of existing data, and which pertain to suicide prevention but may not necessarily involve evaluation of suicide prevention initiatives. Suicide prevention research covers a broad range of research types (e.g. epidemiological, intervention and evaluation studies), suicidal behaviours (suicide, attempted suicide, etc.), all target groups (age, Indigenous, LGBTQI, substance abuse, etc.) and a range of settings (communities, schools, workplaces, mental health services, etc.) [78]
Suicide prevention services	Screening of patients for suicidal ideation, operation of 24/7 hotlines, and engaging all stakeholders for regular follow-up, among other features [79]
Stakeholder	Any group or individual who is affected by or can affect the achievement of an organization’s objectives [80] including policy-makers, service providers and service users/consumers

## Methods

### Approach

Based on the aims of the study, where we are examining levels of stakeholder participation, power differentials and the impact of multisectoral collaborations in evaluation studies, we consider a systematic review to be the most appropriate method [26]. Given this is a mixed-methods systematic review with heterogeneity in study designs, no meta-analysis was undertaken, and instead we conducted a narrative synthesis and applied a quality appraisal [18].

### Identification of studies

#### Search strategy to identify studies

Consistent with best practice, this systematic review was undertaken according to the Cochrane Collaboration Handbook on Systematic Reviews of Health Promotion and Public Health Interventions [27]. The reporting of the results adhered to the guidelines as set out in the Preferred Reporting Items for Systematic Reviews and Meta-Analyses (PRISMA) [28]. No protocol was registered.

#### Search and retrieval of studies

**Fig. 1:**
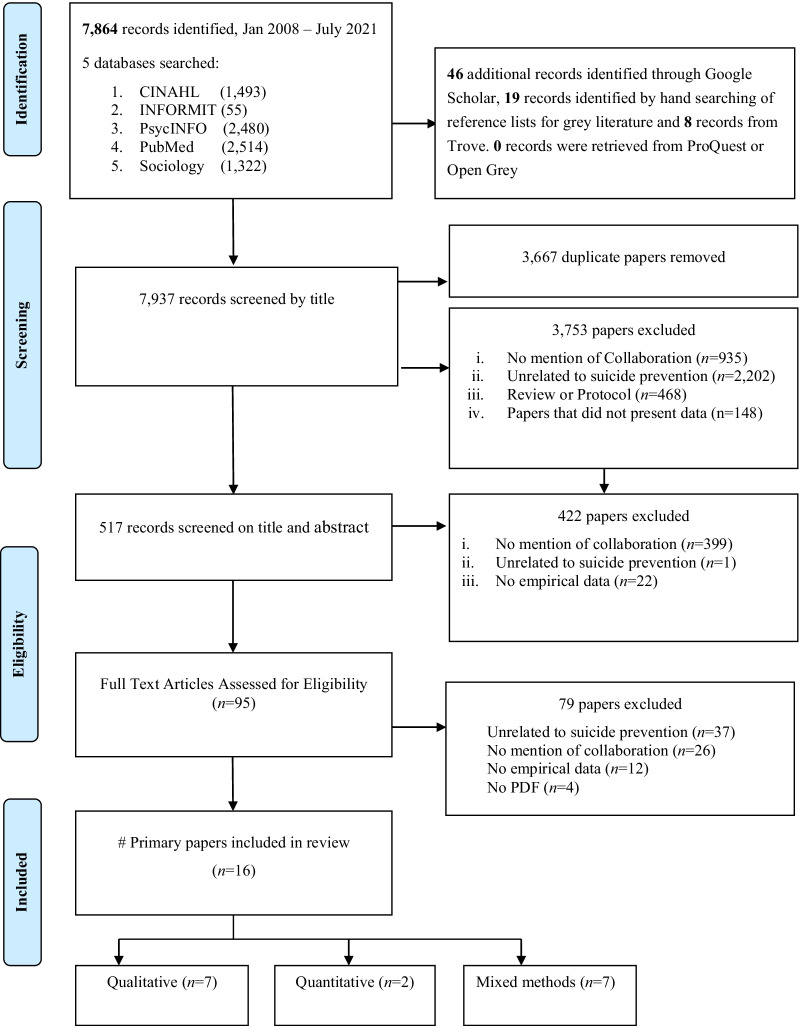
PRISMA flow diagram summarizing systematic search used to identify literature on collaboration in suicide prevention research

Before completing formal searches of the electronic databases, test searches of the databases using a combination of keywords and subject headings were trialled by the lead author. Testing of the search strategy in each of the databases served a dual purpose. First, it provided evidence of which keywords and subject headings would maximize the retrieval of relevant material. Second, the test searches helped to inform the selection of databases. As a result of the pretesting of the search strategy, five scientific electronic databases were identified as providing the highest number of relevant records. On 28 July 2020, TP carried out a search of five bibliographic databases: Cumulative Index of Nursing and Allied Health Literature (CINAHL)/EBSCO, Informit/RMIT University, PsycINFO/ProQuest, MEDLINE/PubMed and Sociology Collection/ProQuest. An additional search of Google Scholar (first 200 records) was undertaken to identify grey literature along with hand-searching of the reference lists of relevant systematic reviews and meta-analyses. The search of the five databases was updated on 6 August 2021 by the lead author. The keywords/Medical Subject Headings (MeSH) terms used to search the electronic databases included three sets of terms: (“Suicide" [Title/Abstract] OR “Suicide” [MeSH] OR “Suicide, Attempted” [MeSH] OR “Suicidal ideation” [MeSH]) AND (“prevention and control” [MeSH] OR “prevention" [Title/Abstract] OR “intervention” [Title/Abstract] OR postvention [Title/Abstract] OR “crisis intervention”) AND (involvement OR participation [Title/Abstract] or collaboration OR engagement OR co-design [Title/Abstract] OR multisectoral [Title/Abstract]. Due to the high rate of false positives, we elected to exclude the keyword “partnership”. Searching “partnership” retrieved records which were focused on the governance of partnerships as opposed to collaboration between researchers and stakeholders. Where appropriate, truncation, wildcards and proximity operators (e.g. co P/0 creation) were used on keywords to maximize the retrieval of relevant records. For instance, participation was truncated as participat* to retrieve keywords such as participation, participating and participatory. We did not search “community based participatory research”, “participatory health research”, “participatory research” or “participatory action research”, as these would be retrieved by default through the search of the keyword “participatory”. In the final stage of the search, the lead author used the same keywords, without language restrictions, to search Google Scholar and other grey literature databases including ProQuest, OpenGrey (grey literature from Europe) and Trove (grey literature from Australia). Reference lists of eligible studies and review studies retrieved through Google Scholar were also hand-searched to identify additional records. For this study, grey documents were defined as unpublished dissertations, government reports, nonprofit programme evaluations, conference proceedings and book chapters. Search results were imported into EndNote X9, a bibliographic management programme, and duplicate citations removed using EndNote’s duplicate identification tool. Rigorous manual checks for any remaining duplicates were also undertaken. Figure 1 describes the search, selection and screening process.

#### Eligibility criteria

The authors relied on five criteria to guide the eligibility process: (i) records with English language abstracts published between January 2008 and July 2021 in either peer-reviewed journals or as grey literature, (ii) papers that described collaborative activities between at least one institution (e.g. university researchers) and one stakeholder group (e.g. citizen/community groups, private entities, TSOs), (iii) papers where the primary focus was on suicide prevention interventions, (iv) papers reporting measurable service, intermediate or health outcomes, and (v) papers containing empirical data (defined as quantitative, qualitative or mixed-methods research). Since this analysis was aimed at assessing the extent of participation by researchers and stakeholders across the research cycle, papers without a clear methodology were excluded. Literature reviews, protocols, news items, editorials or conceptual presentations of untested research models were also excluded. Articles were also removed if multisectoral collaborative activities were incidental to the main research questions, given the specific focus on active, planned collaboration. Where several papers reported on outcomes of the same study (e.g. government report and peer-reviewed journal article), only the most recent was included as the primary source.

#### Study selection

The initial screening of records by title was undertaken by one author (TP). Articles excluded through this process included letters, editorials, news items, and protocol and review papers. Following this, the remaining unique records were exported from EndNote into a Microsoft Excel spreadsheet. Due to the large volume of records, they were divided evenly between two authors (AW and SW), who then independently screened the titles and abstracts according to a pro forma screening instruction sheet. Meanwhile, to ensure accuracy and uniformity, a third author (TP) conducted a blind review of all of the records at the title and abstract stage. Adjudication of disputed records was undertaken by MM* and KM.

#### Data extraction

Papers chosen for full-text review were screened by TP and KM, with basic data extraction of quantitative, qualitative and mixed-methods studies performed using a data extraction template in Microsoft Excel. Data extracted included (1) aims (copied and pasted from each article), (2) focus of study, (3) setting, (4) country, (5) framework, (6) study type, (7) types of stakeholders, (8) level of involvement, (9) types of activities and (10) outcomes.

#### Risk of bias

The risk of bias was assessed using the current 2018 version of the McGill Mixed Methods Appraisal Tool (MMAT) [29, 30]. The MMAT was developed to allow for the concurrent critical appraisal of quantitative, qualitative and mixed-methods primary research in mixed-methods systematic reviews. This tool has been assessed for both reliability and validity and determined to be adequate [30]. In evaluating studies, the MMAT applies five criteria relating specifically to five study design types (qualitative and quantitative randomized controlled trials, and quantitative non-randomized, quantitative descriptive, and mixed-methods studies). Following this, an overall quality score is calculated for each study by dividing the number of positive criteria met by 5. The quality scores vary from 20% (*), representing one criterion met, to 100% (****), where all criteria are met. Studies were assigned quality scores by two reviewers (SKJ and SW) using the checklist, with any differences resolved through discussion. No studies were excluded on the basis of quality.

#### Level of involvement typology

To the best of our knowledge, no typology capturing the level of involvement of stakeholders specific to the research cycle currently exists for health research. Levels of engagement in systematic reviews are commonly assessed using typologies which capture levels of community participation (e.g. Pretty’s typology [31]). However, as the aims of this study were to assess the level of involvement of stakeholders across the research cycle and the value of their participation, the authors chose to use a typology adopted from environmental research. This typology, which describes eight categories of stakeholder engagement, has been empirically tested and validated [32] and is suitable for application in other research fields (Table 2). In addition to the stakeholder typology, the authors applied the co-creation of new knowledge definition [9] to highlight the involvement of stakeholders in four specific stages (co-ideation, co-design, co-implementation and co-evaluation) of the research cycle.

**Table 2:** Typology of stakeholders

Type of stakeholder	Definition
Initiators	Responsible for initiating or driving research proposal
Shapers	Involvement is at the early stage of the research planning process, e.g. consolidating the research plan or providing support or direction
Informants	Assume a consultancy or gatekeeper role informing a research study through focus groups, secondary data, interviewees, etc.
Central	Assume multiple roles including shapers, informants, reviewers, etc., and have vested interest in the research, often participating in advisory groups
Reviewers	Contribute to the shaping and review of outputs
Recipients	Those not directly involved but have an interest in the findings
Reflectors	Provide feedback on research methods or ideas for future research
In-directs	Wider stakeholders who may unconsciously contribute to the research

#### Outcomes framework (service, intermediate social and health)

Outcomes were classed as service, intermediate social and health-related outcomes. These categories were based on a model of community engagement proposed by Popay [18] where service outcomes represent intervention processes, intermediate social outcomes include social effects and empowerment at the community level, and health outcomes are defined as positive or negative changes in individual health. Central to this framework is the idea that as community participation increases from consultation and co-production to empowerment and community control, so does the impact on service, intermediate social and health outcomes. This model has been cited by studies on community participation [33, 34].

## Results

### Study selection

As summarized in Fig. 1, 7937 records were identified from the systematic search of five electronic databases and key grey literature sources. After removal of 3667 duplicates, 4270 records remained. In the first stage of the screening by title, 3753 records were excluded, with the main reasons for elimination being unrelated to suicide prevention, representing those that did not contain empirical data including review papers, protocols, editorials or commentary. The second stage of screening based on title and abstract led to 422 articles being excluded because there was no coverage of collaboration activities or partnerships or they were unrelated to the topic of suicide prevention. Of the remaining 95 potentially eligible papers, full-text screening was undertaken, with another 79 papers being excluded for failing to meet the inclusion criteria. A further two papers were initially assessed as meeting all of the criterial however, as they contained minimal detail about collaborative activities, it was decided to exclude these records from inclusion in the review. Sixteen articles thus met the review inclusion criteria.

### Characteristics of included papers

Of the final 16 papers included in the systematic review, there were seven qualitative [35–41], seven mixed-methods [42–48] and two quantitative studies [49, 50]. The majority of the studies were conducted in HICs, including the United States [38–40, 48–50] and Australia [36, 41, 44–46], with two papers originating from LMICs [37, 47]. Most studies took place in communities [36–42, 44–46, 48–50]. Eight of the articles used a community-based participatory research (CBPR) framework [36, 38–41, 43–46, 49, 50]. The included publications were published between 2009 and 2020 (Table 3).

**Table 3:** Key Characteristics and outcomes of studies

First author, year of publication (country) Publication type	Aim(s)	Setting	Framework	Study type	Outcomes	MMAT score (%)
Allen, 2009 (United States) [49] Journal article	Evaluate changes in community readiness to engage in suicide and alcohol prevention activities and to build protective factors for youth	Community	CBPR	Quasi-experimental	Increase in community readiness and significant (*p* < 0.05) increase in number of protective behaviours in youth (*Intermediate social outcome*)	60
Braun, 2020 (Austria) [35] Journal article	Explore the experiences of adolescents producing suicide prevention videos targeting other adolescents in a school setting	School	N/A	Qualitative	Youth experienced personal growth; felt more capable of safely communicating about suicide, understanding feelings of suicide and offering support. Involvement of young people in the development of prevention material led to creation of materials targeting the needs of peers (*Service outcome*)	100
Brown, 2020 (Australia) [36] Journal article	Describe a qualitative study to increase understanding of how a mobile application could be used to support suicide prevention gatekeepers	Community	Participatory research	Qualitative	Gatekeepers should be trained across multiple roles, and apps should include culturally appropriate refresher content; information on accessing peer support and debriefing and be available across multiple platforms (*Service outcome*)	100
Bruck, 2018 (United States) [48] Thesis	Examine the process of conducting a project with young people as co-researchers, and factors associated with youth mental health	Community	N/A	Mixed methods	Participating in research process included increases in psychological empowerment. No changes reported on measures of self-esteem. Examination of factors associated with mental health in young people (higher reported depression, anxiety, and self-harm and suicidal behaviours). Prefer to seek help from friends when experiencing emotional problems (*Intermediate social outcome*)	60
Chaniang, 2019 (Thailand) [47] Journal article	Develop, implement and evaluate a suicide prevention programme	Community	Action research	Mixed methods	Significant enhancements in the post-test mean scores of suicide knowledge and attitude among adolescent peer leaders, parents and schoolteachers (*Intermediate social outcome*)	40
Chowdhury, 2013 (India) [37] Journal article	Develop a deliberate self-harm suicide prevention programme	Community	CBPR	Qualitative	Based on admission data, the project led to a reduction in deliberate self-harm (*Health outcome)*	100
Ford-Paz, 2015 (United States) [38] Journal article	Understand limitations of existing targeted suicide prevention programmes; identify sociocultural factors leading to the development of depression in Latino adolescents; generate ideas for culturally tailored depression/suicide prevention interventions	Community	CBPR	Qualitative	Culturally tailored suicide prevention interventions may be more effective in reducing depression and suicide in Latino adolescents and utilizing non-mental health professionals and strengths-based strategies may help to decrease depression in at-risk Hispanic young people (*Service outcome*)	100
Gryglewicz, 2014 (United States) [39] Journal article	Provide an example of how to partner with community participants when producing a community-based suicide prevention resource	Community	CBPR	Qualitative	Partnership between community participants and researchers promoted a co-learning and empowering environment (*intermediate Social outcome)*	100
Holliday, 2018 (United States) [40] Journal article	Determine a pilot project and test a pilot project to prevent suicide and substance use	Community	CBPR	Qualitative	Community identified that project addressing youth suicide and substance abuse was priority; project should be cultural and strengths-based and should use readily available resources (*Service outcome*)	100
Le & Gobert, 2015 (Canada) [42] Journal article	Engage in translation of a mindfulness curriculum for cultural relevancy and conduct a feasibility study of the curriculum with a sample of Native American youth	Community/School	N/A	Mixed methods	Mindfulness intervention is acceptable to Native American youth, with positive indications in terms of better self-regulation, less mind-wandering and decreased suicidal thoughts (*Intermediate social outcome*)	0
Mullany, 2009 (United States) [50] Journal article	Examine suicide and suicide attempt rates and risk factors	Community	CBPR	Quantitative descriptive	Universal and targeted suicidal behaviour prevention interventions may be most effective when started within younger age groups; successful implementation of community surveillance system (*Service outcome*)	60
Nasir, 2017 (Australia) [41] Journal article	Review existing gatekeeper training programmes and identify key elements	Community	CBPR	Qualitative	Limitations of existing gatekeeper training programmes included irrelevance, inconsistent content and unsustainable for rural and regional Indigenous communities. Key elements identified for culturally appropriate gatekeeper training programmes included short duration, practical, relevant to language, sustainable, adaptable across communities, cost-effective, integrate existing resources with a holistic focus on community well-being (*Service outcome*)	80
O’Grady, 2020 (Ireland) [43] Journal article	Develop a mobile app to facilitate service users’ access to mental health support and safety planning	Clinical	Participatory research	Mixed methods	Creation of a mental health app with the main benefits being overall design and privacy and data security features (*Service outcome*)	40
Povey, 2020 (Australia) [44] Journal article	Draft a culturally appropriate e-mental health resource	Community	Participatory research	Mixed methods	Apps may overcome barriers by increasing mental health literacy, providing anonymity and linking users with support. Preferred app elements included a strength-based approach, mental health information, relatable content, fun and appealing, with user-friendly interface (*Service outcome*)	80
Skerrett, 2018 (Australia) [45] Journal article	Describe the design and implementation of a group-based intervention and report on measures	Community	CBPR	Mixed methods	Decrease in suicidal ideation, psychological distress and low self esteem; improved understanding of holistic health and coping skills, due to involvement and acceptance by local community, increased referrals to organizations providing mental health support (*Health outcome*)	100
Thorn, 2020, (Australia) [46] Journal article	Aim to document key elements of the co-design process, to evaluate the co-design process and to document recommendations	Community	Participatory research	Mixed method	Increase in suicide literacy among young people; co-design process was feasible, safe and acceptable (*Service outcome*)	60

### Multisectoral collaborations

Six different groups of stakeholders were identified: (i) citizens and communities, (ii) clinical staff, (iii) researchers, (iv) TSOs, (v) government agencies and (vi) private organizations (Tables 4, 5). The most common type of stakeholders found to collaborate with suicide researchers included citizens and communities (87%, 14/16) [35–37, 39–42, 44–50]. In the TSO group there was a diverse range of stakeholders collaborating with researchers including mental health [39, 45], drug and alcohol [41], community services [41], family violence [41], youth [38], suicide prevention [41] and postvention organizations [41]. The group of stakeholder types least likely to be involved in partnerships with researchers included TSOs (37%, 6/16) [37–39, 41, 44, 45] and government bodies (25%, 4/16) [41, 44, 45, 49].

**Table 4:** Types of stakeholders and level of involvement in multisectoral collaborations

First author, year	Types of stakeholders (Reimbursement)	Activities*	Co-ideation	Co-design	Co-implementation	Co-evaluation
			Pre-implementation		Implementation	Post-implementation
Allen, 2009 [49]	1,3,5	Participated in focus groups to provide feedback on interview tools; community planning group facilitated by researchers developed programme and programme module activity	N/A	*R, I*	N/A	N/A
Braun, 2020 [35]	1,3	Co-created 7 short videos and participated in qualitative interviews	N/A	*I*	N/A	*I*
Brown, 2020 [36]	1,2,3	Attended workshops, provided feedback on appropriate apps	*I*	*I*	N/A	N/A
Bruck, 2018 [48]	1,2,3	Lay researchers participated in all 5 phases of the research process, participated in a process evaluation providing feedback	*C*	*C*	*C*	*C*
Chaniang, 2019 [47]	1,3	Generated ideas in discussion groups, advised on programme design and content, facilitated implementation, critiqued programme feasibility	*I*	*R, I*	*C*	*I*
Chowdhury, 2013 [37]	1,2,3,4,6	Participated in focus group to develop communication material and training modules; formulated and endorsed community intervention plan	*S*	*I*	N/A	*R*
Ford-Paz, 2015 [38]	2,3,4	Involved in choosing project topic, conducting focus groups, attended research meetings, recruited participants, participated in coding and analysis of qualitative data, presented results ideas to study participants, sought feedback from participants on research findings	*C*	N/A	*C*	*C*
Gryglewicz, 2014 [39]	1,3,4	Participated in advisory committee; identified and recruited research participants, discussed community concerns and needs, developed and reviewed educational materials, reviewed coding of qualitative data	*S, R*	*C*	N/A	*R*
Holliday, 2018 [40]	1,3	Determined the project topic, involved in grant writing, consulted on culturally appropriate terminology, attended research meetings, participated in events to promote project, oversaw whole of the project with university members, developed recruitment inclusion criteria and recruited participants, analysed data for accuracy	*C*	*I*	*C*	*C*
Le & Gobert, 2015 [42]	1,3	Provided feedback and approval on project proposal, served as tribal champion connecting researcher to tribal agencies, reviewed curriculum; provided feedback on modules, developed research design, protocol, obtained ethics approval	*S, R*	*R*	*R*	N/A
Mullany, 2009 [50]	1,2,3	Educated community on the surveillance system and trained stakeholders on how to complete forms, data processing, discussed research results and developed suicide prevention strategies, developed and standardized data collection process, reviewed research paper for publication	N/A	*C*	*C*	*C*
Nasir, 2017 [41]	1–5	Consulted on the review of gatekeeper training packages, assessed the content of gatekeeper programmes for cultural appropriateness, checked the validity and relevance of the research outcomes	N/A	*I*	N/A	*R, I*
O’Grady, 2020 [43]	2,3	Designed app, implementation and tested the product	N/A	*I*	*I*	*R, I*
Povey, 2020 [44]	1,3–5	Participated in advisory group, provided advice on engagement, recruitment, data collection, analysis and drafting of the resource	N/A	*I, R*	*C*	N/A
Skerrett, 2018 [45]	1–5	Provided advice and approved programme, participated in advisory group and steering committee, provided cultural governance, attended monthly meetings, met with evaluation team, participated in data collection and discussions on measures to use, provided feedback	N/A	*R, I*	*C*	*I*
Thorn, 2020 [46]	1,3	Develop social media campaign, test prototype	N/A	*I*	N/A	N/A
Total frequency of stakeholder types across research cycle			*8*	*15*	*9*	*11*

*The number of individuals participating in each activity varied, and not all participants were involved in every activity

Types of stakeholders: 1 = citizen/community, 2 = clinical staff, 3 = researcher, 4 = TSO, 5 = government, 6 = private entity

Stakeholder role: *R* reviewer, *I* informant; *S* shaper, *C* central

**Table 5:**
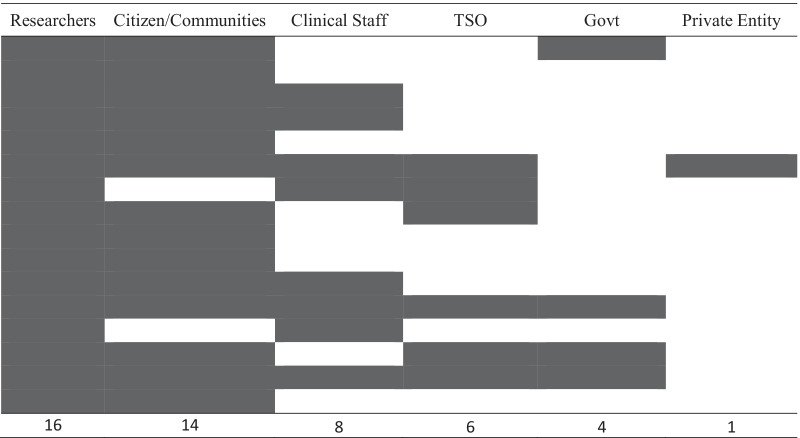
Stakeholder collaboration

### Level of involvement across the research cycle

Based on the descriptions of the research activities contained in each of the included studies, one study provided evidence of stakeholders playing a significant “central” role in all four stages of a research cycle [48]. Participation in the research cycle varied across the remaining three stages (Table 4).

### Pre-implementation stage (co-ideation/co-design)

In the vast majority of papers, activities took place in the pre-implementation stage—otherwise known as the co-ideation and/or co-design phase—of the research cycle (*n* = 23). During this early stage, the bulk of activities involved stakeholders acting as “reviewers” providing feedback on a variety of research instruments including interview protocols [49], participant recruitment criteria [40, 44], curriculum modules [42] and data collection methods [44]. Meanwhile, stakeholders adopting an “informant” role acted as consultants in the design of suicide awareness campaigns [35], educational materials for families [39], the recruitment of participants for community assessment and planning [40], content for secondary school suicide prevention programmes [47, 48] and testing of social media prototypes [46].

### Implementation (co-implementation) and post-implementation (co-evaluation) stage

There were a combined 19 instances of stakeholder participation in the co-implementation and co-evaluation or post-research phase. In this stage, the most common roles adopted by stakeholders included “informant” [35, 41, 43, 45, 47], “reviewer” [37, 39, 41–43, 47] and “central” stakeholder [38, 40, 44, 45, 48, 50]. In this end stage, stakeholders were involved in evaluation activities ranging from assessment of programme feasibility [47] and providing feedback on data collection measures [45], to completing process evaluations [48] and checking the validity and relevance of outcomes [41]. Across included studies, stakeholder types initiators, recipients, reflectors or in-directs were not identified.

### Outcomes

All of the included studies reported positive outcomes (see Table 2). Sixty-two percent (*n* = 10/16) reported on *service outcomes* such as an increase in community suicide literacy amongst young people [46], creation of an app tailored to mental health service users [43] or drafting of an e-mental health resource for Indigenous communities [44]. *Intermediate social outcomes* only accounted for four papers (25%) [39, 42, 48, 49], where participation in the research process increased psychological empowerment [48] and partnership promoted a co-learning environment [39] and increased community readiness towards suicide prevention activities [49]. Only two CBPR studies reported statistically significant *health outcomes*, with Chowdhury et al. [37] finding a reduction in deliberate self harm following implementation of a programme on preventing suicides by pesticide, while a group-based social emotional well-being intervention reported a decrease in suicidal symptomology [45]. In applying Popay’s [18] model of engagement to gauge impact on outcomes according to level of involvement across the research cycle, four studies indicated the potential for high impact on either service outcomes [38, 40, 50] or intermediate service outcomes [48]. Those studies where stakeholders were involved as an informant or reviewer may have had little impact on service [35, 36, 41, 43, 46], intermediate social [42] or health outcomes [37].

### Risk of bias of included studies

Results of the assessment of the risk of bias are included in Table 3. MMT ratings varied according to study design (0–100%). Of the 16 identified studies, over 56% (*n* = 9/16) were rated as being of high quality, scoring 80% or more. The six studies scoring below 75%, and therefore rated as being of moderate to low quality (as per MMAT guidelines), included mixed-methods (*n* = 4); quasi-experimental (*n* = 1) and quantitative approaches (*n* = 1). Qualitative studies (*n* = 7) were methodologically stronger than quantitative and mixed-methods papers. Only two of the seven mixed-methods studies were rated as high-quality. The more common limitations for quantitative studies were the representativeness of the sample used and inappropriate measures [49]. In some of the mixed-methods studies, the quality of reporting was compromised by poor integration between the different methods and inadequate explanation of differences between quantitative and qualitative data [43, 45]. No studies explicitly reported on their philosophical underpinnings or the inherent tensions that can arise when conducting mixed-methods studies. Only one paper made note of the challenge of integrating scientific approaches into community-based research [42].

## Discussion

The aim of this systematic review was to review the current state of evidence on multisectoral collaborations in suicide prevention research. In doing so, we explored the levels of engagement, how power was distributed, outcomes of multisectoral collaborations, and the methodological quality of studies. Sixteen studies met the inclusion criteria. Of these, only one unpublished thesis provided comprehensive evidence of participation by stakeholders across all research stages [48]. Our systematic search of the literature, covering a 13-year period, indicated that research reporting of multisectoral collaborations between suicide researchers and stakeholders has only come about during the last decade. However, growing interest from WHO [2] and national government policies on multisectoral collaboration is a call to action for suicide prevention researchers and stakeholders to consider this type of collaboration as an innovative approach for increasing the impact of research working alongside vulnerable population groups [23, 51]. This review makes a valuable contribution to understanding the impact of multisectoral collaborations on the research cycle and the service and health outcomes of suicide prevention research stemming from these types of collaborations. From the included papers, stakeholders are recruited from a variety of settings and participate at varying levels across the research cycle. Overall, our review indicates that stakeholders remain on the fringes of multisectoral collaborations. A majority of stakeholders were assigned to the role of informant (*n* = 15) or reviewer (*n* = 9) during the co-ideation and co-design stage of the research cycle, where they were involved in providing feedback on planning of programme evaluations. This is consistent with the evidence from previous systematic reviews, where stakeholders were more likely to be involved in the pre-implementation (co-ideation and co-design) phase of the research cycle than in the implementation (co-implementation) and post-implementation stages (co-evaluation) [24, 25]. Prior studies on public and patient involvement in health research have reported similar findings [33, 52], reinforcing the idea that the co-ideation and co-design stages may be an important entry point for stakeholders (consumers and service providers, etc.) to ensure their needs are aligned with the outcomes of research and programmes [53, 54].

Participation in the pre-implementation phase not only provides a reflection of policy focus on the co-design phase of programme evaluation but may also be an indicator of stakeholder interest around the aspects of the programme implementation process affecting them directly [55]. While stakeholder participation in the pre-implementation phases as consultants allowed for researchers to source “insider knowledge” from stakeholders, providing user value to the research planning, design and development of culturally tailored interventions [35, 36, 41, 42, 49], it did not permit stakeholders to fully participate in the decision-making process, and thus researchers appeared to retain more powerful positions in the research cycle in terms of decision-making around the overall planning and management of activities. Also, as theorized in some community engagement models [18], the minimal involvement of stakeholders identified in half of the included studies could indicate that the impact of outcomes was marginal. These findings suggest that the objective of generating meaningful and applicable research through stakeholder participation in the research process may not always be achievable. Time constraints, inadequate funding and lack of resources appear to affect the realization of equality between researchers and stakeholders. Also, the different interests of different stakeholders can be incompatible given their different primary tasks (that is, service providers primarily need to deliver services and researchers primarily need to produce scientifically rigorous research [56], while consumers are focused on satisfactory services). This divergence of interests between researchers and stakeholders could lead to distrust and hamper meaningful engagement by stakeholders in the research cycle. However, as suggested by Israel et al. [15], it is impractical to apply principles of equal engagement to all partnerships, as these will be dependent on the community or culture in which the research is taking place. Multisectoral collaborations between researchers, TSOs and government, for instance, might involve different group dynamics and power differential when compared to collaborations involving only researchers and community members.

It is also recognized that differing levels of engagement will depend on the makeup of the stakeholders involved in the multisectoral collaboration [15]. While this might appear to “limit” stakeholder contribution (e.g. consultative), it does not mean that all stakeholders need to be involved equally across the research cycle [57]. In fact, the notion of sharing equal power across all aspects of participatory research may not be the best approach for optimizing evaluation outcomes. This could certainly be the case in multisectoral collaborations involving long-term research projects where role distribution and group dynamics between researchers and stakeholders are likely to change over time [58]. As suggested by Bryson et al. [57], identification and analysis of stakeholders according to their level of influence, power, expertise and interest in either the programme or the evaluation can be of benefit by enhancing meaningful stakeholder engagement while also contributing to programme legitimacy, credibility, usefulness and cost-effectiveness [57, 59]. The challenge in achieving stakeholder equity highlights the inherent complexities of using participatory research methods in multisectoral collaboration. As noted by other participatory researchers [54, 60], this issue may remain unresolved; however, what is clear is the importance of researchers conducting regular assessments of power relations when working in multisectoral collaborations and ensuring that the level of involvement allows them to impact on agreed outcomes [61].

The review also uncovered issues with methodological rigour and intervention research. Although the quality appraisal of the studies found a majority to be methodologically appropriate, the choice of research methodology was predominately qualitative, with the purpose of gathering data from end-users to inform the development of suicide prevention interventions. Explanations for the increased use of qualitative methodology include (1) the high number of studies relying on participatory frameworks favouring reflective and iterative processes, which is compatible with qualitative methodologies compared with quantitative studies, and (2) the call from suicide researchers to increase our understanding of the suicide experience through qualitative studies, which may have also contributed to this increase in qualitative research [62]. Advancing the use of multisectoral collaborations and improving the evidence base for suicide prevention research in general may require the consideration of frameworks and approaches such as co-creation of new knowledge where rigorous research methods are embedded in the delivery of the programme [9]. Surprisingly, in this review, there were fewer studies using mixed-methods research designs even though these types of approaches offer the benefits of multiple perspectives and are therefore well suited to understanding complex problems such as suicide [63]. Our findings corroborate those of Salimi et al. [64], who suggested that collaborative studies involving researcher–stakeholder partnerships often lack scientific rigour [64]. The appraisal of evidence in this review revealed evidence of small sample sizes [38, 44, 45] and inadequate explanation of statistical analysis provided in mixed-methods studies [45] and basic analysis of results [43]. Although the combination of rigorous methods and participatory research may appear incompatible, it does not necessarily mean a loss of rigour or relevance [65]. Increased commitment and support from stakeholders regarding the use of rigorous research methods and embedding these into the delivery of programmes or policies of TSOs will help to build a quality evidence base, capacity and sustainability over time.

## Strengths and limitations

To our knowledge, this is the first study to consider the extent to which suicide prevention researchers are engaging in multisectoral collaborations with consumers and other stakeholders. Also, the reporting of this paper has been strengthened by input from service providers and consumers of mental health services and stakeholders from TSOs. The screening process was systematically carried out by two reviewers using a well-defined screening tool to ensure uniformity when applying the eligibility criteria. The study has several limitations. First, although the authors searched multiple databases using a targeted search strategy, it is acknowledged that some relevant papers may have been missed. When searching for evidence of consumers or stakeholders, the lack of standardized terminology to categorize these groups within the literature is problematic. To counteract this problem, the search strategy was deliberately designed to be broad in order to retrieve all papers on multisectoral collaboration in the field of suicide prevention regardless of whether they involved consumers with lived experience, specific population groups such as Indigenous or culturally and linguistically diverse (CALD) populations, or mental health services delivered by TSOs or other community-based organizations. Further to this, the authors’ choice of limiting grey literature searches to developed countries may have introduced some geographical bias. The decision to search OpenGrey and Trove was based on the authors’ understanding of an increasing trend and government support for multisectoral collaborations in suicide prevention in both Europe and Australia. Preliminary searches of the grey literature in LMICs failed to identify relevant records. This finding is perhaps a reflection of the political and social barriers existing around suicide prevention in LMICs, which has limited the overall number of studies published [66]. Finally, our study was limited by the minimal number of studies on multisectoral collaborations in the field of mental health and suicide prevention. The low number of results in our papers mirrors the findings of two earlier papers on participatory involvement in mental health research [24, 25]. Such small numbers may be indicative of the practical challenges researchers and stakeholders face when undertaking participatory research in multisectoral collaborations compared with other fields of study, such as psychology, with participatory methods representing less than 1% of papers when compared to peer-reviewed studies published in mainstream psychology journals [67].

### Implications for service delivery and policy implementation

For service delivery and policy implementation, this systematic review offers insight into the current state of play regarding collaboration in the field of suicide prevention research. TSOs are an important stakeholder, especially their need for evaluation of the quality and effectiveness of the services and programmes they offer [68]. Their input into the research process is essential for improving the quality and effectiveness of their services, for instance, developing new ways of improving services or developing new research techniques [68]. Yet, despite the general understanding that TSOs are an important provider of the delivery of suicide prevention services, our findings show TSOs were less likely to be involved in multisectoral collaborations with researchers. Furthermore, with government policy supporting multisectoral collaborations, this raises the question as to what barriers are preventing engagement between researchers and TSOs. Or if, TSOs are participating in specific co-creation processes such as co-design and co-ideation, why are they not researching and writing about it? Or further, if they are participating with researchers in these early phases, why they are not included throughout the process or in the authorship? Research suggests that one reason for this may be linked to the organizational mission statement and culture where the needs of their clients are prioritized over engagement in research and evaluation [69]. With regard to consumers with “lived experience”, of the evidence that is available in this review, there is no indication this group had assumed a lead stakeholder role in suicide research. This is in contrast to government policy recommendations supporting the engagement of consumers and broader partnerships in the design and development of suicide prevention research to help tailor solutions to meet the needs of those with lived experience [4, 70, 71]. This finding highlights the need to close the policy/implementation gap through genuine engagement among all stakeholders, including consumers, using frameworks such as co-creation of new knowledge [9]. Finally, it would be useful for researchers to document who provided funding for these participatory projects, as this would allow targeting of these groups for co-creation of knowledge and improving the quality of what is being funded.

## Implications for Future Research

This discussion has provided several key areas requiring attention for future research. There is an assumption that participation by stakeholders in multisectoral collaborations would lead to a higher standard of research outputs. However, in this systematic review, regardless of the level and depth of engagement by a variety of stakeholders, the methodological quality in over half of the studies, while appropriate, was weak. This is comparable to the quality of evidence produced by traditional research approaches [72, 73]. We recommend the investigation of whether, over time, improved approaches to multisectoral collaborations lead to increased use of rigorous research methods, a core principle of co-creation of new knowledge [9]. In order to draw clear conclusions about the effect of engagement on research quality, suicide prevention researchers need to provide detailed descriptions of activities undertaken by stakeholders across the research cycle and the depth of engagement. Future research should also consider at minimum implementing sound research design such as quasi-experimental or experimental research methods to advance the empirical basis of suicide prevention studies involving multisectoral collaboration. Ensuring the impact of multisectoral collaborations on the efficacy of suicide prevention interventions should be considered to develop an enhanced understanding of the relationship between multisectoral collaboration and suicide prevention research output. Further, monitoring the level of participation by stakeholders to understand the extent and quality of multisectoral collaboration warrants further investigation. While there is evidence of increased interest in multisectoral collaborations in LMICs, suicide remains highly stigmatized, and in some countries illegal [74]. We suggest that future research systematically document processes for developing best practice models for multisectoral collaborations in LMICs while considering the local sociocultural environment [51].

## Conclusion

Overall, the findings of this systematic review reveal that multisectoral collaborations demonstrate some promise in improving the evidence base, although increased attention is needed on supporting effective interventions through the use of appropriate and robust study designs. The review also found that the level of stakeholder engagement as consultants in the planning stages of the studies limited their opportunities to shape the programmes and interventions. It further identified that dimensions of power are explicitly and implicitly embedded in multisectoral collaborations through levels of engagement and the language used to describe stakeholders. For the field of suicide prevention, academic studies on multisectoral collaborations appear to indicate a growing acceptance of the importance of using innovative approaches for complex issues. Suicide prevention is a field of knowledge that is evolving rapidly, and collaboration between researchers, policy-makers, service providers and communities is integral to reducing suicide deaths and associated morbidity. Multisectoral collaborations remain a valuable and worthwhile endeavour with the potential to produce meaningful and sustainable research which in turn can lead to increased transfer of knowledge into practice and reduction of research waste. However, collaboration should not be undertaken simply for its own sake. It is imperative to ensure that the collaboration will lead to effective interventions by meeting the needs of stakeholders. While time and funding barriers impact on the practice of equality within researcher/stakeholder collaboration, it is crucial that researchers acknowledge the skills and expertise stakeholders can offer to the research cycle. Collaboration has the potential to improve experiences for people who use programmes and services, and this requires an understanding of their needs and preferences, for their feedback to be valued by researchers so that it may be translated into service improvement and utilized as the basis for evaluation measures and frameworks.

## Data Availability

The data used to support the findings of this study are available on request from the corresponding author.

## Abbreviations

CBPR: Community-based participatory research
CALD: Culturally and Linguistically Diverse
IKT: Integrated knowledge translation
LMICs: Low- and middle-income countries
PRISMA: Preferred reporting items for systematic reviews and meta-analyses
TSOs: Third-sector organizations
